# Change in convergence and accommodation after two weeks of eye exercises in typical young adults

**DOI:** 10.1016/j.jaapos.2013.11.008

**Published:** 2014-04

**Authors:** Anna M. Horwood, Sonia S. Toor, Patricia M. Riddell

**Affiliations:** a Infant Vision Laboratory, School of Psychology & Clinical Language Sciences, University of Reading, United Kingdom; b Orthoptic Department, Royal Berkshire Hospital, Reading, United Kingdom

## Abstract

**Background:**

Although eye exercises appear to help heterophoria, convergence insufficiency, and intermittent strabismus, results can be confounded by placebo, practice, and encouragement effects. This study assessed objective changes in vergence and accommodation responses in naive young adults after a 2-week period of eye exercises under controlled conditions to determine the extent to which treatment effects occur over other factors.

**Methods:**

Asymptomatic young adults were randomly assigned to one of two no-treatment (control) groups or to one of six eye exercise groups: accommodation, vergence, both, convergence in excess of accommodation, accommodation in excess of convergence, and placebo. Subjects were tested and retested under identical conditions, except for the second control group, who were additionally encouraged. Objective accommodation and vergence were assessed to a range of targets moving in depth containing combinations of blur, disparity, and proximity/looming cues.

**Results:**

A total of 156 subjects were included. Response gain improved more for less naturalistic targets where more improvement was possible. Convergence exercises improved vergence for near across all targets (*P* = 0.035). Mean accommodation changed similarly but nonsignificantly. No other treatment group differed significantly from the nonencouraged control group, whereas encouraging effort produced significantly increased vergence (*P* = 0.004) and accommodation (*P* = 0.005) gains in the second control group.

**Conclusions:**

True treatment effects were small, significantly better only after vergence exercises to a nonaccommodative target, and rarely related to the response they were designed to improve. Exercising accommodation without convergence made no difference to accommodation to cues containing detail. Additional effort improved objective responses the most.

Orthoptic exercises for convergence insufficiency, heterophoria, and intermittent strabismus have been in use for over 70 years.^1^ They may involve intensive, clinic-based vision training or simpler, home-based, exercises. Orthoptists have generally adopted less intensive methods over the decades, while “vision therapy” textbooks and some branches of optometry continue to support intensive therapy.^2^ The lack of strong evidence was identified by the Convergence Insufficiency Treatment Trial (CITT) Group in designing a large multicenter trial^3^ comparing the effects of different treatment regimens on convergence insufficiency, a condition where most professionals agree that exercises are effective. Studies clearly suggest that children receiving office-based therapy had the best outcome^4^, ^5^, ^6^, ^7^, ^8^, ^9^, ^10^ compared to home-based methods; however, despite great efforts in the study design, true treatment effects and particularly the added benefit of therapist encouragement on simple exercises, could have accounted for apparent additional improvements in patients receiving intensive office therapy. Recently, Fray^11^ has remarked on the many uncertainties and inconsistencies concerning the effects of different testing methods and instruction sets among clinicians as well as the level of alertness in participants.

Research concentrates on relief of symptoms,^3^, ^12^ but symptomatic improvement could also be due to placebo effects, without changes in ocular responses. It is therefore unclear how exercises influence *objective* changes in accommodation and convergence. Some evidence suggests that accommodation and proximity responses are less susceptible to training than convergence^13^, ^14^ and that vergence change may mediate accommodation change.^15^ It is unknown whether accommodation exercises improve only accommodation, convergence via the accommodative convergence/accommodation (AC/A) linkage, or neither, and whether exercises that stress vergence in excess of accommodation (or vice versa) are more effective than those that stress them in their natural relationship, or than those that only stress one system.

This study aimed to assess objective changes produced by short courses of different exercises, with particular attention to the relative influence of practice, placebo, and encouragement effects, which are not well researched, even in normal populations.^15^, ^16^, ^17^, ^18^ In view of the paucity of normative data by which to judge the CITT and other vision therapy studies, we performed a baseline study on typical young adults. Our previous work^19^ found that disparity drives the majority of convergence *and* accommodation. Accordingly, we expected that exercises based on enhancing the response to disparity would be more effective than those based on blur resolution. We also have evidence that hypo-accommodation is common for naturalistic targets if clarity is not stressed; thus we predicted that exercises stressing the importance of clarity might improve accommodation.^19^

## Methods

This study adhered to the Declaration of Helsinki, and no objections were raised by the University of Reading Research Ethics Committee. Full methodological details are provided in e-Supplement 1 (available online at jaapos.org) and summarized below.

Participants were recruited from university students 18-25 years of age who considered themselves to have “normal” eyes, aside from spectacles < ±4.00 D. None had a history of past ocular treatment or had taken part in prior vision research. Volunteers were excluded if they had significant ocular symptoms (≥16 on an adjusted CISS questionnaire^7^) and if orthoptic examination revealed any abnormality, such as manifest strabismus, exophoria >6^Δ^ or esophoria >1^Δ^, any vertical deviation, convergence poorer than to 8 cm, or a low fusion range (<25^Δ^ base-out and 10^Δ^ base-in for near). Participants were informed that we were investigating how different types of eye exercises affected focusing in comparison to practice/repetition effects. They were not told that we were also investigating placebo treatment and effort effects.

Two testing sessions were carried out 10-18 days apart, with 98% of tests exactly 2 weeks apart and at the same time of day. Testing was carried out wearing any current refractive correction (glasses or contact lenses). At both visits, participants were tested by the same researcher, who was masked to the treatment group allocation, except for those in the effort group, who were given extra encouragement, so masking was not possible. Scoring of laboratory data was carried out masked to participant identity and treatment group allocation.

### Remote Haploscopic Photorefractor

**Fig 1:**
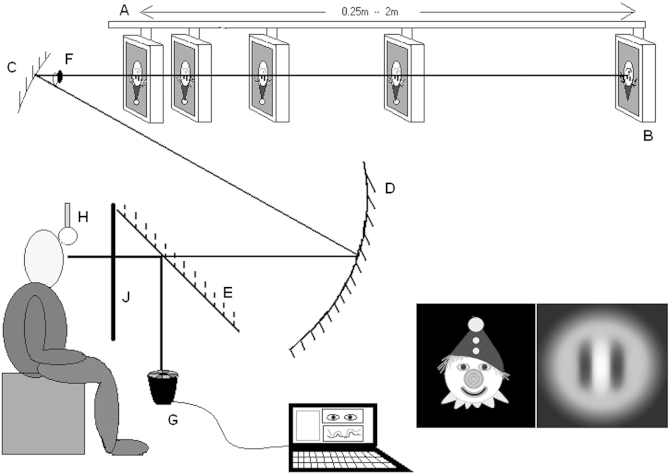
The remote haploscopic videorefractor. The two targets (brightly colored clown target or Gabor image were designed to maximize or minimize blur cues, respectively)*. A*, motorized beam. *B*, target monitor, moving between fixation distances. *C*, upper concave mirror. *D*, lower concave mirror. *E*, “hot” mirror. *F*, image of subject's eye, where occlusion takes place. *G*, PlusoptiX S04 PowerRef II. *H*, headrest. *J*, black cloth screen, which can be raised to occlude the target when required.

The examination method has been described in detail elsewhere.^19^ Briefly, all participants watched the target being presented on a video monitor via a two-mirror optical system, while a PlusoptiXS04 PowerRefII photorefractor (Plusoptix GmbH, Nuremberg, Germany) collected simultaneous eye position and refraction measurements (Figure 1).

We could manipulate blur (B) by using a detailed clown cartoon or a blurry Gabor patch target to present or minimize detail cues. Disparity cues (D) were available by allowing binocular fixation, or could be prevented by occluding half of the upper mirror. Proximity/looming (P) cues were available if the participant watched the same target moving between fixation distances, or could be minimized by screening the monitor as it moved and scaling the target so that it subtended the same visual angle at each position. Thus 8 different target conditions representing all combinations of presence or absence of these cues were possible (Table 1). All other aspects of the data collection and testing paradigm were identical.

**Table 1:** Target cue conditions. Target presented in the different cue conditions: *b*, blur present in cue; *d*, disparity present; *p*, proximity/looming present; *o*, minimal cue condition

Stimulus	Target		
	Disparity	Blur	Proximity
Blur + proximal + disparity (bdp)	Both eyes open	Clown	Unscaled
B removed (dp)	Both eyes open	Gabor	Unscaled
D removed (bp)	Occluded	Clown	Unscaled
P removed (bd)	Both eyes open	Clown	Scaled
B only (b)	Occluded	Clown	Scaled
D only (d)	Both eyes open	Gabor	Scaled
P only (p)	Occluded	Gabor	Unscaled
None (o)	Occluded	Gabor	Scaled

Instructions were minimal so that we could assess responses in as naturalistic manner as possible. In the all-cue, naturalistic (BDP) condition many asymptomatic participants would be expected to show excellent responses, with little room for improvement (a ceiling effect), at least for vergence. But as cues were removed, we expected to be able to detect more changes in the reduced accommodation and vergence responses that these “impoverished” targets typically produce, and that these responses might be specific to the exercise regime. We wanted to determine whether an exercise targeting just blur or just disparity helped responses to accommodation or vergence differentially or overall. On each visit measurements were repeated twice in a counterbalanced order, with an orthoptic testing session between the measurement periods.

Accommodation response in diopters and vergence in meter angles were calculated from the raw data, corrected for measured angle lambda, interpupillary distances and any spectacle magnification.

### Exercises

After the initial testing session, each participant was randomly allocated to one of 8 experimental groups by a second researcher masked to test results (Table 2). If exercises were given they were to be done 3 times daily for 5 minutes.

**Table 2:** Exercise regimens carried out three times a day for five minutes

Group	Skill manipulated	Target	Exercise	Subject end point
Blur	Accommodation only; blur independent of disparity	Detailed	Monocular push-ups Monocular near /distance “jump” accommodation Monocular accommodation facility (+2/−2D [near], 0/−2D [distance] lens flippers)	Blur
Both	Accommodation and convergence in normal relationship	Detailed	Binocular push-ups Binocular “jump” vergence/accommodation Near/distance physiological diplopia	Blur or diplopia
Disparity	Vergence independent of accommodation	Gabor image	Binocular push-ups Binocular “jump” vergence Near & distance vergence facility (12^Δ^ BO/4^Δ^ BI prism flippers)	Diplopia
Conv+	Convergence in excess of accommodation	Detailed	Binocular push-ups (+2.0 D or 12^Δ^ BO) Binocular near accommodation facility(0/+2.0 D) Binocular near & distance vergence facility (0/12^Δ^ BO)	Blur or diplopia
Accom+	Accommodation in excess of convergence	Detailed	Binocular push-ups (−2.0 D or 12^Δ^ BI) Binocular near and distance accommodation facility (0/−2.0 D) Binocular near (and distance if possible) vergence facility (0/12^Δ^ BI)	Blur or diplopia
Motion (placebo)	Attention, motion detection, proprioception	Visual illusions; physical objects	“Snakes illusion”: max/ min moving Necker cube: perceptual shift Yoked prisms: visually directed reach with /without prisms	
Nil	Practice, test–retest		None	
Effort	Tester, instruction set, effort		None	

Orthoptic exercises were administered specifically to target: (1) blur, that is, blur awareness and accommodation but not disparity awareness or vergence; (2) both, or use of maximal vergence and blur awareness in a balanced (naturalistic) relationship; (3) disparity, that is, vergence and disparity awareness independent of blur/clarity; (4) “conv+,” that is, convergence in excess of accommodation (positive relative convergence or negative relative accommodation); (5) “accom+,” that is, accommodation in excess of convergence (positive relative accommodation or negative relative vergence); or (6) motion, or placebo “treatments” that did not exercise the vergence or accommodation systems, involving attention, motion detection, and proprioception. There were two no-treatment groups: (7) “nil,” to assess practice and repetition effects; and (8) “effort,” a no-exercise group that at the second testing session was exhorted to maximum effort.

Participants in each group were told what their exercises were for, shown how to do them, and asked to demonstrate to the researcher what they had been taught. The importance of honesty in reporting missed exercise sessions was stressed and diaries and cell phone alarms were used to aid adherence to the protocol.

### Analysis

Data analysis was carried out using Excel and SPSS version 18 (SPSS, Chicago, IL). Three-way mixed ANOVA was performed with cue (8 levels) and response (vergence or accommodation) as within-groups factors and treatment group (8 levels) as a between-groups factor. Post hoc testing used two-way ANOVA and *t* tests with appropriate correction for multiple comparisons. In view of the multiple measures we obtained, in this paper we report the change in calculated convergence and accommodation response gain between first and second testing sessions as well as responses in meter angles and diopters at 33 cm, which was the fixation distance where most changes were found. A gain of 1.0, and 3 meter angles of convergence and 3.0 D of accommodation at 33 cm, indicate appropriate responses to target distance.

## Results

Data from 156 participants were analyzed, and each group included 17-21 participants; 14 additional participants were excluded because they showed evidence of mild convergence insufficiency according to the strict CITT criteria,^4^ despite denying any symptoms, and a further 2 were excluded because lid or eyelash configuration prevented collection of photorefraction data. Two participants who admitted by email that they had not done any exercises were re-allocated to one of the no-treatment groups without breaking the masking of the tester to group allocation.

Informal examination of exercise diary sheets showed no systematic differences between groups, although some individuals had been more assiduous in completing them than others. As objective confirmation of adherence to the regimes could not be further verified, we were unable to analyze this further.

There were no statistically significant differences in range of refractive errors, heterophoria or initial fusion ranges between the 8 groups. Reduced gain (flatter stimulus/response slope) can be due to poor response for near or overresponse in the distance (or both). To investigate this, we analyzed the responses at 33 cm and 2 m. No differences at 2 m approached significance (*P* > 0.4 in all comparisons); thus the changes in gain represent alterations in responses to the closer targets.

**Fig 2:**
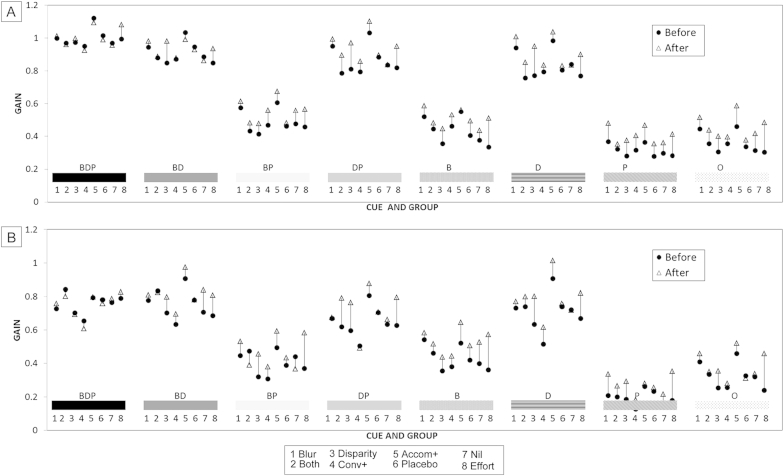
Response gain changes. Change in mean response gain according to cue condition (shaded sections on x-axis) and exercise group (lower text on x-axis) within cue condition A gain of 1.0 indicates perfect performance for target demand. A, Vergence gain change. In all cases above, reduced gain is due to underperformance for near. B, Accommodation gain change. *B*, blur available; *D*, disparity available; *P*, proximity/looming available; *O*, minimal cue condition.

Figure 2 illustrates that most improvements in gain occurred for targets containing fewer cues. The majority of the participants performed near optimally (at ceiling) to the all-cue BDP condition even before exercises, with a mean vergence gain of 1.00 (±95% CI, 0.04) and vergence response of 3.01 meter angles at 33 cm. There was concurrent accommodative lag of 0.73 D at 33 cm, with mean accommodative gain of 0.75 (±95% CI, 0.05). Before treatment, mean vergence and accommodation gains were reduced to all other targets. Therefore, the gains in response to these targets had more potential to improve with exercise.

**Fig 3:**
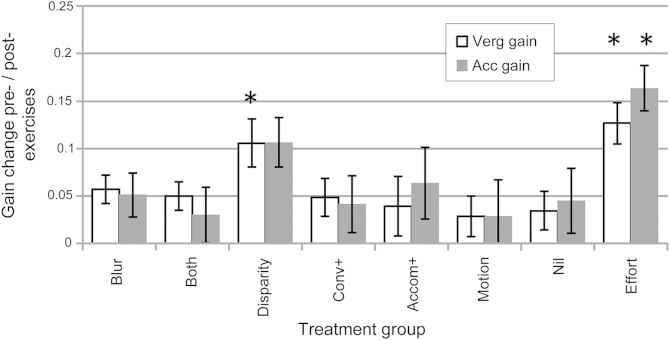
Mean improvement in gain across all cue conditions for the different treatment groups. Error bars denote standard error of the mean. An improvement in gain of 0.1 denotes approximately 0.3 D or 0.3 meter angles at 33 cm (approximately 2^Δ^ for an interpupillary distance of 6 cm). Asterisks denote significant differences from the nil (no treatment) group.

A three-way mixed ANOVA of the within-subjects improvements showed significant differences between groups (F[7,148] = 3.29, *P* = 0.003) as well as the differences between cues which we typically find in all our studies (F[7,148] = 4.75, *P* = 0.0002), and a significant cue × group × response interaction (F[49,148] = 1.4, *P* = 0.04). Post hoc testing showed that, averaged across all the cues, both vergence and accommodation gain improved (F[5,115] = 3.94, *P* = 0.002) and (F[5,115] = 3.42, *P* = 0.006) and were not significantly different from each other (paired *t* test [155] = 0.53, *P* = 0.5). There was wider variance in accommodation change, reflecting the more variable accommodation responses overall (between visits and between and within individuals). Figure 3 shows mean improvement in convergence and accommodation gain averaged across all the cues in the different treatment groups.

The small improvements in the nil group (practice/repetition effect) were then used as the baseline by which to judge the additional effects of treatment or effort.

Only the disparity and effort groups showed statistically significant differences from the nil group. The disparity group vergence gain (*t*[37] = 2.19, *P* = 0.035) improved, but although the mean improvement in accommodation gain was similar, accommodation increases (particularly in the nil group) showed more variance and so differences did not reach significance (*t*[37] = 1.20, *P* = 0.24). Disparity exercises improved vergence responses by an average across cues of 0.35 meter angles (12%) and accommodation by 0.27 D (9%) at 33 cm.

The no-treatment effort group showed the greatest improvement in both vergence and accommodation (*t*[38] = 3.10, *P* = 0.004 for vergence, and *t*[38] = 2.95, *P* = 0.005 for accommodation). Mean vergence across all cues improved by 0.34 meter angles (11%) and accommodation by 0.46 D (15% of the total demanded by the target) at 33 cm, with the most effect seen for the more impoverished targets where responses were poorer at first.

**Fig 4:**
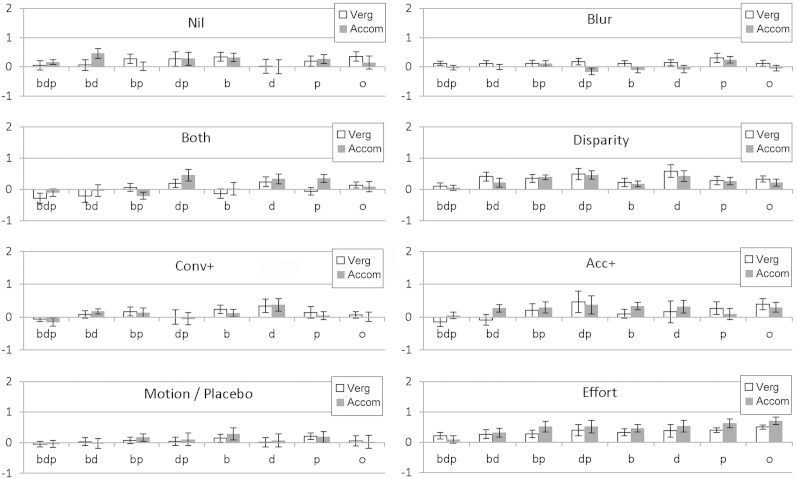
Change in responses at 33 cm after treatment. Convergence in meter angles (1 = approximately 6^Δ^ for average adults); accommodation in diopters. Stimulus: *bpd*, blur + proximal + disparity; *bd*, proximal removed; *bp*, disparity removed; *dp*, blur removed; *b*, blur only; *d*, disparity only; *p*, proximal only; *o*, none.

Figure 4 illustrates the actual changes in vergence (in meter angles) and accommodation (in diopters) at 33 cm for each group and each cue, which may be of more practical significance to clinicians.

Although improvement in overall responses across cues were statistically significant, when broken down by cue, clear patterns were less evident. Disparity exercises improved vergence in the BD (*P* = 0.02) and DP (*P* = 0.03) conditions and marginally in the D condition (*P* = 0.06), that is, when disparity cues were available to be responded to, whereas they improved accommodation only in the BP condition (*P* = 0.049), when they were not available.

Accommodation exercises (the blur group, when participants had been specifically told to concentrate on clearing images for the past two weeks), did not result in mean accommodation for near improving at all (−0.004 D or −0.1%), with no significant differences between accommodation to the targets where detail was available (BDP, BD, BP, B) and those where it was not (DP, D, P, O). If accommodation exercises are effective we expected to find greatest effect from the blur group in the blur-only B condition (where responses are also typically poor, so with good potential for improvement) but accommodation to this target remained poor (gain improved by only 0.04 to 0.58 and accommodation at 33 cm improved only 0.17 D, remaining poor at 1.6 D). Effort alone, however, improved accommodation gain to this target by 0.21 to 0.57 and accommodation at 33 cm improved by 0.5 to 1.8 D.

When the effort group was compared to the nil group, there were improvements in gain across all cues, and these were most marked in the more impoverished targets, and more for accommodation than vergence (Figure 4), but after correction for multiple comparisons the only individually significant differences were for vergence in the BDP condition (*P* = .02), where 8 of the 21 participants (38%) overconverged by more than 10% and for accommodation gain in the BP (*P* = 0.001) and O (*P* = 0.04) conditions.

Although exercises stressing more convergence than accommodation (positive relative vergence/ negative relative accommodation) would be expected to lead to better convergence gain and responses for near than for accommodation, and exercises stressing accommodation more than convergence (positive relative accommodation or negative relative vergence) would be expected to have the opposite effect, neither strategy made any significant difference over the no treatment (nil) group. Although Figure 4 suggests small changes in the predicted directions, none approached statistical significance.

## Discussion

This study investigated medium-term changes in naturalistic responses produced by 2 weeks of different types of exercises on objective measures of convergence and accommodation in typical young adults rather than on symptoms or clinical measures. It provides a normal dataset by which similar changes in patient groups can be judged.

While exercises appear effective, any good therapy uses motivational, effort, practice, and placebo effects that are difficult to quantify. It is important that health economists, patients, and parents accessing treatment recognize, understand, and identify the relative contribution of these factors. For example, the CITT trials^4^ showed that 35% of patients improved with office-based placebo therapy, indicating that placebo and encouragement effects were significant. The additional advantage that the CITT found of in-office therapy could be due to the additive effects of patients being taught the importance of effort in addition to the eye exercises themselves rather than the intrinsic superiority of the more specific or intensive therapeutic techniques. In our study, simple vergence exercises, independent of accommodation, were the most effective therapy; more complex manipulations of vergence and accommodation were less effective.

Unsurprisingly, the greatest treatment effects of vergence practice were found for the BD, DP, and D targets where disparity was available. These targets used reduced cues that produce reduced responses, and therefore, improvement was still possible. Mean accommodation improved as much as vergence with these exercises, and the failure to find statistical significance may be because there was more variability and slightly higher accommodation in the second visit, particularly for the control group.

Any treatment effect of concentrating on resolving blur was very small, if any. The largest improvement was found for the blurred P and O targets, so even when detail was available in any cue using the clown target (BDP, BD, BP, B), practicing and concentrating on clearing images made no difference to naturalistic responses to this detailed target. Blur exercises, placebo exercises, and no treatment had very similar effects.

The conv+ and accom+ and the both groups, all stressing concentration on clarity *and* single vision, did not produce better, or as good, responses as found in the disparity group, where clearing the target had been impossible. In our lab we repeatedly find that disparity is by far the strongest driver of responses. Thus finding that disparity exercises were the most effective was not surprising; however, it is unclear why stressing fusion *independent* of accommodation is better than exercising clarity and fusion. Although some exercises were possibly less demanding or less realistic than others, we attempted to devise exercises demanding a similar amount of effort.

Even in this asymptomatic, typical, young adult population, where vergence in the most naturalistic condition was good (near ceiling), we still produced treatment effects, but they were small and often not very different from those found by just repeating the same tests on a second occasion. Effects in patient groups might of course differ, but this study provides a baseline with which to compare them. In clinical groups, where values are outside normal ranges, treatment-induced changes are likely to be greater. It is also possible that our treatment period was too short for changes in naturalistic behavior to be detected or that children might behave differently from adults.

We were not able to prove that participants had complied with the exercises, but there appeared to be no systematic between-group differences. Study participants were mostly science undergraduates, who would presumably appreciate the value of both obtaining and providing accurate data. They were told that we could still use their data if they had not practiced, as long as we knew before the second testing session. We also said that we expected that the laboratory data would tell us if they had cheated, although we were less confident that this would be the case.

The test–retest variability of the no-treatment control group (particularly for accommodation) may have hidden subtle effects. All groups except this nil group were encouraged to use some element of effort or attention either while doing exercises or during second testing, so might have been more consistent in their change in responses, as the overall patterns suggest (Figure 4), while the nil group received no instruction. Levels of effort on the second visit in the nil control group could have varied more due to familiarity (less effort) or practice (more effort), respectively. Adler and colleagues^18^ found considerable inter-tester and within-subject accommodative variability, presumably tapping in to similar effects, in primary school children.

It is clear that the greatest influence in changing responses to an approaching target is how the participant was instructed and the amount of effort they exerted. This effect was more noticeable when there was more room for improvement for reduced-cue targets where habitual responses were poorer. Many individuals seemed happy to leave images blurred unless told to try to clear them. While this may not surprise those who deliver any form of therapy, if a specific exercise is to be assessed for effectiveness, then instructions and levels of effort expected should be identical before and after treatment. It may also be important that levels of alertness be assessed. Fray^11^ has reported similar effects of encouragement in the testing of convergence amplitudes and found that lower levels of alertness affected fusion ranges. The additional benefit of in-office vision therapy is likely to be due to the patient getting more encouragement and reinforcement to try harder. Any claims for specific treatment effects must be considered in relation to this. In view of the importance of effort in comparison to true treatment effects of different exercises and the costs in terms of professional time, loss of schooling, and many office visits of a long course of in-office vision therapy, maximizing motivation and feedback strategies for less costly home exercises seems desireable.

## Supplementary Data

e-Supplement 1
