# Supplementary material for: Change in convergence and accommodation after two weeks of eye exercises in typical young adults
Source: J AAPOS. 2014 Apr;18(2):162–8. doi: 10.1016/j.jaapos.2013.11.008 (PMC3991418; doi:10.1016/j.jaapos.2013.11.008)
Supplement: e-Supplement 1 [file mmc1.pdf]

## e-Supplement 1

### Participants

Participants were young adults (18-25 years of age) recruited from the School of Psychology Participant Panel and from advertisements circulated within the University. A primary inclusion criterion was that they "considered themselves to have normal eyes (apart from weak glasses < ± 4.00 DS)". Any volunteer who had a past or current strabismus, amblyopia or CI, who had sought advice for a visual symptom, or who had prior orthoptic exercises given by an optometrist or orthoptist, was not eligible. Participants were also ineligible if they had already taken part in any other research in our laboratory.

Most participants were first and second year psychology undergraduates with some knowledge of basic behavioral experimental methodology such as masked studies, randomization and placebo effects, but no more vision science knowledge than the general population. A small additional number were undergraduates and post-graduates from other academic disciplines unrelated to medicine or optics. All were managing a typical academic workload.

Two laboratory testing sessions were carried out two weeks apart (minimum 10 days –maximum 18 days). 98% of participants were tested exactly 14 days apart and at the same time of day. Testing was carried out wearing any current refractive correction, either spectacles or contact lenses. If spectacles were worn, a correction for magnification effects in the calculation of the vergence response were made based on previous unpublished calibration studies. Testing before and after treatment was always carried out wearing the same type of correction i.e. glasses or contact lenses.

Participants were told that the experiment was to investigate how different types of eye exercises affected the objective responses in the laboratory and that we were interested in the relative strength of different treatment effects in comparison to practice/repetition effects. They were not told that we were also investigating a placebo treatment and tester instruction/ encouragement effects. They were told that there was controversy in the field and that we had no *a priori* predictions about what the study would find. Participants were tested by the same experimenter on both visits, who was masked to the treatment group allocation, except for those in the "encouragement" group. Scoring of laboratory data was carried out masked to participant identity and treatment allocation.

Participants were asked to fill out the Convergence Insufficiency Symptom Survey (CISS) questionnaire [1]to exclude ocular symptoms. In view of the participants' typical academic workload and student lifestyles, supplemental questions were asked for items 4, 5, 6, 9 and 15 to ascertain if any symptoms (such as feeling sleepy, having to re-read text or difficulty remembering what they had read) were ascribed to an ocular cause or not. If they did not ascribe the symptoms to an ocular cause, the question was scored as zero. Results from this part of the study have been reported separately (under submission to JAAPOS). If they scored ≥16 on this adjusted scale they were excluded from the analysis. A brief history was taken to confirm absence of ocular problems and that any spectacles were a current prescription (within one year). Particular attention was paid to maintaining a standard testing protocol and instruction set in the laboratory with all tests carried out in the same order. A full set of accommodation and convergence data (see Photorefractor section) were then collected to all eight cue conditions.

After this testing, a full orthoptic investigation was carried out. We modeled our testing protocol as much as possible on the CITT Protocol and Manual of Procedures[2,3] in terms of tests, testing order and test administration methods. For the clinical tests such as accommodation and convergence near point and prism fusion ranges participants were told to watch the target and “try hard to keep the targets clear or single (as appropriate for the test) for as long as possible”, and to tell the experimenter when they were unable to maintain clarity or single vision any longer. The experimenter used a standard, positive, friendly, professional tone of voice. The only specific encouragement was to say “well done“ or “good” at the end of each test. Results of these clinical tests will be reported separately, but if any showed clinical signs of convergence insufficiency such as convergence to 8 cm or poorer, or a fusion range not fulfilling Sheard’s criterion (a fusion range of more than double the heterophoria)[4], the participants were excluded from this study.

The full laboratory photorefraction sequence was then repeated in a counterbalanced testing order and the data were averaged. For testing with the photorefractor the participants were just instructed to “watch the target.” The testing protocol was identical on the second visit to the laboratory.

**Remote Haploscopic Photorefractor**

The method has been described in detail elsewhere,5 but briefly all participants watched the target being presented on a video monitor via a two-mirror optical system, while a PlusoptiXSO4 PowerRefII photorefractor collected simultaneous eye position and refraction measurements (Figure 1). The whole apparatus was encased in matte black shuttering and targets moved between five different fixation distances (0.33 m, 2 m, 0.25 m[1], 1 m, 0.5 m) in the same pseudo-random order each time.

We could manipulate blur (B), disparity (D) and proximity/looming (P) cues separately, while all other aspects of the data collection

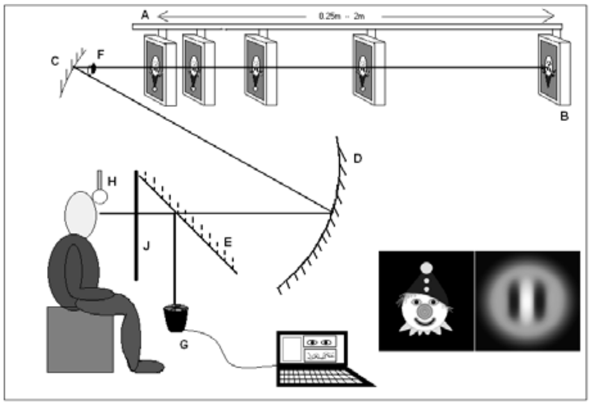


**Figure 1**

and testing paradigm were identical. Blur cues could be presented by using a complex cartoon clown target containing a wide range of spatial frequencies and detail down to 1 pixel (<1 arcmin), or could be minimized by using a blurred Gabor image to open the accommodation loop as much as possible while retaining fusible features for the binocular conditions. The Gabor image had a maximum spatial frequency of 1.58 cycles per degree. Unpublished prestudy trials with targets of lower spatial frequency did not further open the accommodation loop but did present a poorer fusional stimulus. Disparity cues were available when both eyes could view the target, and could be eliminated by occluding half of the upper mirror (C in Figure 1), so that the target was then only visible to one eye but data were collected by the photorefractor from both eyes via the lower optical pathway. Proximity and looming cues were available when the target remained the same size on the screen and could be watched as it moved backwards and forwards

[1] The data from this target position were discarded for technical reasons not associated with the study.

| Stimulus | Target | | |
|---|---|---|---|
| | **Disparity** | **Blur** | **Proximity** |
| Blur + Disparity + Proximal *(bdp)* | Both eyes open | Clown | Unscaled |
| B removed *(dp)* | Both eyes open | Gabor | Unscaled |
| D removed *(bp)* | Occluded | Clown | Unscaled |
| P removed *(bd)* | Both eyes open | Clown | Scaled |
| B only *(b)* | Occluded | Clown | Scaled |
| D only *(d)* | Both eyes open | Gabor | Scaled |
| P only *(p)* | Occluded | Gabor | Unscaled |
| None *(o)* | Occluded | Gabor | Scaled |

Table 1.

(subtending between 3° and 18° depending on distance), or these dynamic and size cues could be minimized by scaling the target so that it subtended the same retinal angle (3°) at each distance and hiding the screen from view with a black curtain while it moved between the five positions. Thus eight target conditions were possible (Table 1) and by using these cue conditions we could assess naturalistic responses (BDP condition) and two different ways of assessing the influence of each cue (firstly, when it was eliminated from a naturalistic situation (BDP vs. BD, BP and DP conditions) as might occur when degraded in suppression or refractive error, and then when it was the only cue presented (B, D, and P conditions). A minimal (O) condition assessed the effect of any residual cues we could not totally exclude. Instructions were minimal so that we could assess responses in as naturalistic manner as possible.

A specially written Excel macro calculated diopters of accommodation (D) and meter angles of vergence (MA) using the raw data spreadsheet produced by the photorefractor. We plotted these data on an intermediate chart from which we selected short vignettes of 25 stable and representative data points (one second of data) at each fixation distance. Corrections were made for measured angle lambda, inter-pupillary distances (IPD), and any spectacle magnification for each participant, all of which can be calculated from individual raw data.[18] This is particularly important for the calculations of vergence in MA that allow comparisons between appropriateness of vergence responses between participants with different IPDs. Accommodation was assessed as the amount of myopic shift from calculated refraction at infinity (y-intercept of the accommodation response slope in the all cue BDP condition), with a correction for a systematic error compared with dynamic retinoscopy found in calibration studies. We did not carry out individual calibration of the accommodative responses as suggested by Bharadwaj et al [6] for two reasons; firstly our measurements were well within the reported linear operating range of the photorefractor, but mainly because our analyses compared responses within individuals before and after treatment and between cues, so would be independent of between-subjects variability. No participants were significantly anisometropic (<1.00 D in any meridian).

In the all-cue, naturalistic, condition many asymptomatic participants would be expected to perform at ceiling (at least for vergence), but in the impoverished cue conditions we expected to be able to detect more changes in the (reduced) accommodation and vergence responses, and that these might be specific to the exercise regime, e.g. if an exercise targeting just blur or just disparity helped

responses to accommodation or vergence differentially, or overall.

## Exercises

At the end of the initial testing session each participant was randomly allocated to one of eight experimental groups by a second experimenter masked to any test results. Six of the groups involved doing homework exercises for five minutes three times a day for the subsequent two weeks, and the other two groups were told they were in the control/repetition group and did not have to do any exercises (Table 2).

Orthoptic exercises were given specifically to target i) blur awareness and accommodation, but not disparity awareness or vergence; ii) use of maximal vergence *and* blur awareness in a balanced (naturalistic) relationship; iii) vergence and disparity awareness independent of blur/clarity; iv) convergence in excess of accommodation (positive relative convergence / negative relative accommodation) v) accommodation in excess of convergence (positive relative accommodation / negative relative vergence) vi) placebo "treatments" that did not specifically exercise the vergence or accommodation systems but which involved attention, motion detection and proprioception. Because of evidence that response to disparity is a major drive to accommodation, exercises targeting blur perception alone needed to be carried out monocularly in order to be sure that response to disparity was not an additional influence.

## Detailed exercise regimes

1. BLUR EXERCISES. Monocular blur awareness (accommodation exercises) using a highly accommodative target (Figure 2a) for near fixation and a detailed distance fixation target such as leaves on a tree. If they had a smartphone they were also allowed to use the tiniest images in the app folder icons, which gave a similar amount of fine detail. We did not use a binocular task because without complex lens/prism combinations we would not be able to differentiate blur-driven from disparity-driven accommodation if improvement occurred.

   They were told that the target must be kept <u>clear</u> at all times, and to alternate which eye they used for fixation.

   a) Monocular near point push-ups using an accommodative target

   b) Monocular "jump" accommodation from distance to 6-10cm from nose and vice versa maximising the speed of clearing the image.

   c) Monocular accommodative facility practice using +2.0/−2.0 D flipper lenses at 33 cm and 0/−2.0 D at distance (>6 m) fixation (counting "flips" per minute, only moving to the next "flip" once the target was clear).

2. "BOTH" EXERCISES. Accommodation & convergence together in a naturalistic relationship using similar targets to the Blur group (Figure 2a), stressing they should be kept clear *and* single at all times.

   a) Binocular convergence and accommodation push-ups, with the near point being recorded when it either blurred or went double.

   b) Jump convergence and accommodation between near (6-10cm) and distance targets, trying to increase the speed of response, also stressing clarity and fusion throughout

   c) Practice appreciating physiological diplopia using the accommodative target for the fixation target (Figure 2a) and a high contrast lower spatial frequency image (Figure 2d) for the non-fixated "diplopic and blurred" target both behind and in front of the fixation target. The participant was to

concentrate on awareness of how the fixation target remained clear and single but the non-fixated target was both blurred and double.

| Group | Skill manipulated | Target | Exercise | Subject end point |
|---|---|---|---|---|
| Blur | Accommodation only; blur independent of disparity | Detailed | Monocular push-ups<br>Monocular near /distance “jump” accommodation<br>Monocular accommodation facility (+2/−2D [near], 0/−2D [distance] lens flippers) | Blur |
| Both | Accommodation and convergence in normal relationship | Detailed | Binocular push-ups<br>Binocular “jump” vergence/accommodation<br>Near/distance physiological diplopia | Blur or diplopia |
| Disparity | Vergence independent of accommodation | Gabor image | Binocular push-ups<br>Binocular “jump” vergence<br>Near & distance vergence facility (12$^{\Delta}$ BO/4∆ BI prism flippers) | Diplopia |
| Conv+ | Convergence in excess of accommodation | Detailed | Binocular push-ups (+2.0 D or 12$^{\Delta}$ BO)<br>Binocular near accommodation facility(0/+2.0 D)<br>Binocular near & distance vergence facility (0/12$^{\Delta}$ BO) | Blur or diplopia |
| Accom+ | Accommodation in excess of convergence | Detailed | Binocular push-ups (−2.0 D or 12$^{\Delta}$ BI)<br>Binocular near and distance accommodation facility (0/−2.0 D)<br>Binocular near (and distance if possible) vergence facility (0/12$^{\Delta}$ BI) | Blur or diplopia |
| Motion (placebo) | Attention, motion detection, proprioception | Visual illusions; physical objects | “Snakes illusion”: max/ min moving<br>Necker cube: perceptual shift<br>Yoked prisms: visually directed reach with /without prisms | |
| Nil | Practice, test–retest | | None | |
| Effort | Tester, instruction set, effort | | None | |

Table 2.

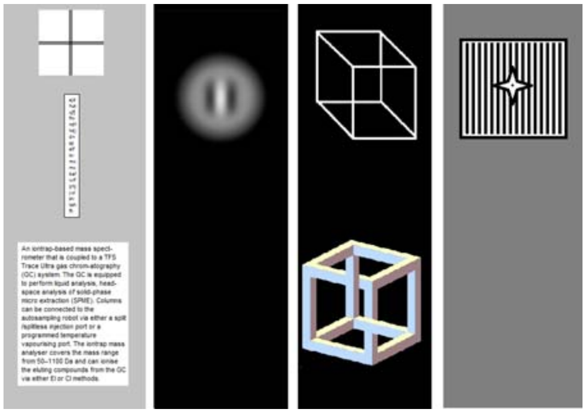

a b c d

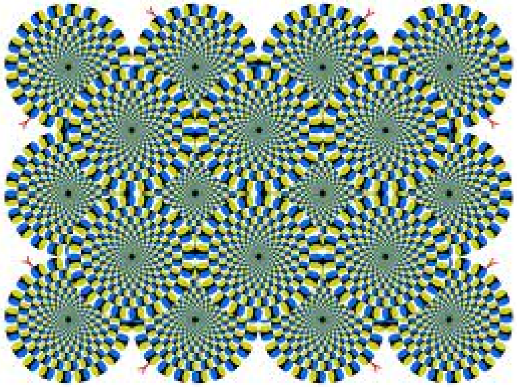

e

*Figure 2. Exercise fixation targets. a) Maximum detail target. Complex small text and a cross consisting of six intersecting lines, such that there were four tiny squares at their intersection. b) Gabor target similar to lab target, minimising detail available but retaining fusible disparity information c) Necker cube targets d) Near or distance image to be used with target a) to appreciate the blur and diplopia of physiological diplopia e) “snakes” illusion image.*

3. DISPARITY EXERCISES. Disparity awareness independent of accommodation using an image of a Gabor patch similar to that used in the laboratory as the fixation target (Figure 2b) to minimise near detail cues while retaining a fusional stimulus. The participants were told that the target should be kept single but was meant to be blurry and so they would not be able to make it clear.
   a) Binocular convergence push-ups.
   b) Binocular “jump” convergence from distance/near and near/distance fixation maximising speed of fusing image
   c) Vergence facility (maximising the number flips per minute) using 12BO and 4BI flipper prisms for both near and distant fixation, moving on to the next “flip” once single vision was attained.

4. CONVERGENCE+ EXERCISES. These were all carried out using the detailed fixation card (Figure 2a) or smartphone icon. Clear and single vision was stressed throughout. They were given a set of flippers containing a pair of +2.0D lenses and pair of 12Δ base out prisms (6Δ each eye). When looking through the lenses at any target vergence would be appropriate but with less accommodation required. When looking through the prisms at any target, accommodation would be appropriate while additional convergence would be required.

   a) Gradual push-ups towards the nose and gradual withdrawal of the target while looking through the lenses and then the prisms.
   b) Lens flippers facility (with lens / without lens, maximum cycles per minute) for near fixation, stressing clarity and fusion before the next “flip”.
   c) Prism flippers facility for near and distance fixation, again stressing clarity and fusion before the next “flip”.

5. ACCOMMODATION+ EXERCISES. These were also carried out using target 2a, stressing clarity and fusion. They were given a set of flippers containing a pair of -2.0 D lenses and a set of $12^{\Delta}$ base-in prisms ($6^{\Delta}$ each eye). When looking through the lenses more accommodation, but normal vergence would be required, and when looking through the prisms divergence (or less convergence) would be required for a normal amount of accommodation for the target distance.
   a) Gradual push-ups towards the nose and gradual withdrawal of the target while looking through the lenses and then the prisms.
   b) Lens flippers facility (with lens / without lens, maximum “flips” per minute) for near and distance fixation, stressing clarity and fusion before the next “flip”.
   c) Prism flippers facility for near (and distance fixation if possible), again stressing clarity and fusion before the next “flip”.

If a participant in any of the Blur, Disparity, Convergence+ and Accommodation+ groups was unable to clear or join images through the prism / lens strengths above, they were given a weaker pair (1.0 D or 6Δ) to use at first. All participants were asked to concentrate their practice on the exercise they found most challenging to maximise any treatment effects.

6. PLACEBO EXERCISES. Placebo treatment concentrating on attention, motion, proprioception and fixation stability.
   a) The “snakes illusion” (Figure 2e) gives an illusion of motion which is minimised by steady fixation and maximised by unsteady fixation. The participants were asked to try to minimise and maximise the number of “snakes” they could get to move simultaneously, and to try to work out what they had to do to do so.

b) Necker cube illusion (Figure 2c), which can be perceived in opposite 3D positions. The participants were asked to maximise the rate at which they could make the perceptual shift to make the illusion reverse.

c) Yoked prism flipper prisms (one set both prisms base up or base down and the other set both base left/right) to make a mis-match between visually guided reach and proprioception. For near they were asked to touch a physical near target e.g. corner of a table, then put up the yoked prisms and try to touch the same target as quickly and accurately as possible; then remove the prisms and repeat, concentrating on accuracy and speed. As they got used to the visual image shift, accuracy improved. For more distance fixation they were asked to make two steps towards a target and touch it, step back, then repeat with the yoked prisms.

7. NO TREATMENT CONTROLS. For this group the participant knew they were a control, but the experimenter was masked to group allocation. The same testing procedure was repeated two weeks later to assess repetition effects.

8. NO-TREATMENT EFFORT GROUP. Participants in this second of the no-treatment groups were also told they were controls but in this case the experimenter was not masked to their allocation. The tester was told to make particular efforts on the second testing session to give maximum encouragement throughout both orthoptic and photorefractor testing. The participants were specifically asked to try to improve on their last responses in terms of effort and accuracy ("go on," "let's see how much you can do if you try REALLY hard" "try harder," "keep going, you are doing really well" "don't worry if it pulls a bit, keep going"). During testing with the photorefractor they were told throughout testing to try as hard as they could to try to keep the target as clear and single as possible.

Participants in the treatment groups were told which aspect of their visual system the exercises were targeting, but those in the Motion (placebo) group were not told they were in a placebo treatment control group. Participants in each group were shown how to do the exercises and asked to demonstrate what they had been taught back to the experimenter before leaving the lab. They were asked to work hard at the exercises and to try to improve their scores on any quantitative responses such as flipper tasks or near points. The exercise groups were asked to set their cell phone alarms to remind them to do the exercises three times a day and to keep a daily diary of each exercise session, recording data such as near points and flipper counts to maximize compliance. They were told that one of the study outcomes was that we expected to be able to relate the lab results to their exercise diaries and how much they had practiced and improved, so we would be likely to be able to tell if exercises had been missed, but we also expected honesty in reporting if exercises had not been done. All of the participants were science undergraduates or post-graduates and would have been aware of the importance of collecting accurate data for any experiment. The exercise diaries were not seen or collected by the experimenters until all testing was completed.

**Analysis**

Data analysis was carried out using Excel and SPSS v18 software. 3-way mixed ANOVA with cue (8 levels) and response (vergence or accommodation) as within-groups factors and treatment group (8 levels) as a between-groups factor was conducted.

Post hoc testing used 2-way ANOVA and *t*-tests with appropriate correction for multiple comparisons where appropriate. In view of the multiple measures we obtained, in this paper we report the change in calculated convergence and accommodation response gain between first and second testing sessions as well as responses in MA and D at 33 cm, which was the fixation distance where most changes were found. A gain of 1.0, and 3MA of convergence and 3.0 D of accommodation at 33cm, indicate appropriate responses to target distance.

**References**

1. Rouse M, Borsting E, Mitchell GL, et al. Validity of the convergence insufficiency symptom survey: a confirmatory study. Optom Vis Sci. Apr 2009;86(4):357-363.
2. ConvergenceInsufficiencyTreatmentStudyGroup. The convergence insufficiency treatment trial: design, methods, and baseline data. Ophthalmic Epidemiol. Jan-Feb 2008;15(1):24-36.
3. ConvergenceInsufficiencyTreatmentTrialGroup. Convergence Insufficiency Treatment Trial (CITT) Manual of Procedures. 2006; http://optometry.osu.edu/research/CITT/8581.cfm.
4. Millodot M. Dictionary of Optometry & Visual Science. 4 ed. Oxford: Butterworth-Heinemann; 1997.
5. Horwood A, Riddell P. The use of cues to convergence and accommodation in naïve, uninstructed participants. Vision Research. 2008;48(15):1613-1624.
6. Bharadwaj S, Sravani N, Little J-A, et al. Empirical variability in the calibration of slope-based eccentric photorefraction. J Opt Soc Am A 2013;30(5):923-931.
